# Metabolic Disruption Induced by mTOR Signaling Pathway Inhibition in Regulatory T-Cell Expansion for Clinical Application

**DOI:** 10.3390/cells12162066

**Published:** 2023-08-15

**Authors:** Roberto Gedaly, Gabriel Orozco, Alexandre P. Ancheta, Mackenzie Donoho, Siddharth N. Desai, Fanny Chapelin, Aman Khurana, Lillie J. Lewis, Cuiping Zhang, Francesc Marti

**Affiliations:** 1 Transplant Division, Department of Surgery, College of Medicine, University of Kentucky, Lexington, KY 40536, USA; roberto.gedaly@uky.edu (R.G.); orozcog@ohsu.edu (G.O.); alexandre.ancheta@uky.edu (A.P.A.); mackenzie.donoho@uky.edu (M.D.); siddharth.desay@uky.edu (S.N.D.); lillie.lewis@uky.edu (L.J.L.); 2 Lucillle Parker Markey Cancer Center, College of Medicine, University of Kentucky, Lexington, KY 40536, USA; fchapelin@uky.edu (F.C.); aman.k@uky.edu (A.K.); 3 Division of Transplantation, Section for Quality and Biostatistics, College of Medicine, University of Kentucky, Lexington, KY 40536, USA; 4 Alliance Research Initiative (*TILT* Alliance), College of Medicine, University of Kentucky, Lexington, KY 40536, USA; 5 Department of Biomedical Engineering, College of Medicine, University of Kentucky, Lexington, KY 40506, USA; 6 Department of Radiology, College of Medicine, University of Kentucky, Lexington, KY 40536, USA; 7 Flow Cytometry & Immune Monitoring Core Facility, College of Medicine, University of Kentucky, Lexington, KY 40536, USA; cuiping.zhang@uky.edu

**Keywords:** regulatory T-cells, transplantation, cellular therapy, mTOR signaling

## Abstract

Background: Regulatory T cell (Treg) therapy is considered an alternative approach to induce tolerance in transplantation. If successful, this therapy may have implications on immunosuppression minimization/withdrawal to reduce drug-induced toxicity in patients. The aim of this study was to assess the efficacy of the mTORC1/C2 inhibitor, AZD8055, in the manufacturing of clinically competent Treg cells and compare the effects with those induced by rapamycin (RAPA), another mTOR inhibitor commonly used in Treg expansion protocols. Methods: Primary human Treg cells were isolated from leukapheresis product. Cell viability, expansion rates, suppressive function, autophagy, mitochondrial unfolded protein response (mitoUPR), and cell metabolic profile were assessed. Results: We observed a stronger inhibition of the mTORC2 signaling pathway and downstream events triggered by Interleukin 2 (IL2)-receptor in AZD8055-treated cells compared with those treated with RAPA. AZD8055 induced progressive metabolic changes in mitochondrial respiration and glycolytic pathways that disrupted the long-term expansion and suppressive function of Tregs. Unlike RAPA, AZD8055 treatment impaired autophagy and enhanced the mitoUPR cell stress response pathway. Conclusions: A distinct pattern of mTOR inhibition by AZD, compared with RAPA, induced mitochondrial stress response and dysfunction, impaired autophagy, and disrupted cellular bioenergetics, resulting in the loss of proliferative potential and suppressive function of Treg cells.

## 1. Introduction

Cellular therapy with autologous regulatory T cells (Tregs) is emerging as an attractive alternative to induce tolerance in solid organ transplantation (SOT). The current paradigm is that the balance between alloantigen-reactive effector T cells (Tconv) and protective suppressive Treg cells contributes to immune homeostasis and plays a crucial role in the tolerogenic induction and maintenance of the allograft. Standard immunosuppressive regimens are usually based on the combination of calcineurin inhibitors, steroids, and anti-proliferative agents and are intended to tilt the effector/regulatory balance by preferentially targeting the activation of effector cells. Although the introduction of immunosuppressants has resulted in significant improvement of short-term allograft survival, long-term survival and drug-related toxicities remain the most significant challenge in SOT. As a result, new therapeutic strategies are being actively investigated to decrease immunosuppressive side effects while improving long-term allograft and patient survival.

In the past two decades, several clinical trials have demonstrated the safety and feasibility of using adoptive Treg cell therapy to control the damaging effects of excessive immune pro-inflammatory responses in type I diabetes, graft-versus-host disease and SOT [1,2,3,4,5,6]. Due to the low number of circulating Treg cells, ex vivo expansion is a crucial step in producing an adequate yield of Tregs for clinical application.

Efforts to optimize the manufacturing process of clinical grade Treg cells are currently focused on improving the purity of the initial Treg cell population, reducing the expansion times to minimize contamination risks and processing costs, and increasing the potency of the final product. Recent studies reveal the impact of fine-tuning the mechanistic target of rapamycin (mTOR) activity to drive proper T cell function and fate decisions [7,8,9,10,11,12,13,14,15,16,17,18,19]. The different sensitivities of effector and regulatory T cells to mTOR signaling modulation [7] supports the key role of this pathway in the distinct regulation of both cell type responses [8]. Early studies revealed that the inhibition of the mTOR pathway by allosteric rapamycin (RAPA) under normal activating conditions led to a preferential inhibition of conventional T cell (Tconv) proliferation and effector function and enhanced the generation of Treg cells [8,9,10,11]. Based on these results, most current manufacturing protocols rely on the use of RAPA to create culture conditions that promote the selective expansion of CD4^+^/CD25^HIGH+^/FOXP3^+^ Treg cells. However, the plasticity within the PI3K/mTOR/AKT signaling cluster that includes cross-talks, feedback loops, and compensatory mechanisms revealed the complex role of mTOR signaling in the regulation of Treg cells. Paradoxically, mTOR activity is increased in human Tregs and supports Treg proliferation [9,13,14]. Likewise, in mice, the integrity of mTORC1, a key regulator of glycolysis, was reported to be necessary for Treg function [15].

Our laboratory and others have addressed the integration of metabolic cues by the mTOR signaling as a critical determinant of Treg cell function and fate [16,17,18,19,20]. Our findings corroborated the distinct mitochondrial oxidation and glycolytic rates of Treg cells and effector Tconv, revealed the importance of the metabolic balance to preserve the functional integrity of Treg cells, and demonstrated the sensitivity of this tightly regulated balance to allosteric mTOR inhibitors. In this context, we have studied multiple agents with different mechanisms of mTOR signaling pathway modulation for Treg cell expansion [16,17].

In our quest to improve Treg cell manufacturing for clinical application and understanding underlying mechanisms involved in adequate ex vivo expansion, we investigated the phenotypic, functional, and metabolic alterations induced by 5-{2,4-bis[(3S)-3-methylmorpholin-4-yl]pyrido[2,3-d]pyrimidin-7-yl}-2-methoxyphenyl)methanol, commercially known as AZD8055. AZD8055 is a potent and selective mTORC1 and mTORC2 inhibitor with reported antitumor activity in a wide variety of human tumors. Here, we will compare the impact of AZD8055 and RAPA on proliferation rates, suppressive function, cellular stress responses, and metabolic balance in primary human Treg cells.

## 2. Materials and Methods

Experimental protocols were approved by and conducted in accordance with the Institutional Review Board Committee at the University of Kentucky (Protocols 47,098 and 47,343). Written informed consent was obtained from all study participants prior to participation.

### 2.1. Drugs and Compounds

Stock solutions of AZD8055 (provided by AstraZeneca, Cambridge, UK) and RAPA (Miltenyi Biotec, Auburn, CA, USA) were prepared in DMSO (vehicle) and used at the indicated final concentrations in culture medium.

### 2.2. Purification and Activation of Primary Human T cells

CD25^+^ Treg cells were isolated from the leukapheresis product of end stage renal patients as previously described [16,17,18]. Briefly, the leukapheresis product was conjugated for 10 min at 4 °C with the CliniMACS CD25 MicroBeads reagent (Miltenyi Biotec). CD25^+^ cells were isolated in the Clini MACS Plus system (Miltenyi Biotec, software program *Enrichment 3.2*), following the manufacturer’s instructions. Treg cells were identified as CD4^+^/CD25^+^/FOXP3^+^/CD127^−^ and isolated with a purity above 85% throughout the study. Tconv were sorted from PBMCs with the human CD4^+^ T cell isolation kit (StemCell Technologies, Cambridge, MA, USA). For initial stimulation of cells, Treg and/or Tconv cells were placed in culture at a concentration of 10^6^ cells/mL in Treg cell culture medium: TexMACS GMP medium (Miltenyi) supplemented with recombinant human Interleukin-2 (IL2, MACS GMP, Miltenyi, at 500 IU/mL) and MACS GMP ExpAct Treg (beads conjugated to CD28, Anti-Biotin, and CD3-Biotin monoclonal antibodies, Miltenyi) at a bead-to-cell ratio of 2:1. Cells were exposed to treatment conditions of vehicle control (DMSO 0.1%), RAPA (100 nM) or AZD8055 (20 nM) for 60 min prior addition of cell activation supplements. Additionally, half of cell culture media was routinely replaced every three days.

### 2.3. Determination of the Minimal Effective Dose of AZD8055

A proliferation assay with Tconv was used. Cellular proliferation was assessed by evaluating the expression of Ki67 after 24 h culture using intracellular staining and flow cytometer analysis. Cells were exposed to different concentrations of AZD8055 ranging from 0 to 4 µM and compared to cells exposed to RAPA (100 nM). We selected the minimal dose of AZD8055 that caused a similar inhibitory response to Tconv proliferation as RAPA 100 nM.

### 2.4. Proliferation Assay

A 4-day proliferation CFSE assay was used to compare the effect of AZD8055 (minimal effective dose), RAPA (100 nM), and no drug (control) on the proliferation of Treg cells. Treg proliferation was also assessed by manual counting for 12 days under different culture conditions.

### 2.5. Cell Viability and Functional Assays

To assess the effect of AZD8055 (minimum effective dose) on Treg cells, we evaluated Treg viability, phenotype, suppressive function, and signaling of Treg cells exposed to AZD8055, RAPA, or control vehicle (no drug). Viability was determined by Zombie-Violet (BioLegend, San Diego, CA, USA) die exclusion after 4-day culture. Suppressive function was evaluated after three days of treatment exposure and determined by the inhibition of 5 × 10^4^ CFSE-labeled T cell expansion in a 4-day mixed lymphocyte reaction with the addition of varying amounts of drug-treated Treg cells at specified Treg/CFSE-responder cell ratios [16,17,18,19].

### 2.6. Cell Surface, Intracellular Staining and Flow Cytometry Analysis

For cell surface and intracellular labeling, single-cell suspensions were stained with fluorochrome-labeled antibodies listed in the Supplementary Data (Table S1). Activation of signaling pathways was evaluated by intracellular staining of pAKT (as mTORC2 substrate), p4EBP1 (as mTORC1 substrate), pSRC (as proximal T cell receptor activation substrate), and pSTAT5 (as IL2-receptor substrate) after 4 days of drug exposure. Flow cytometry data were acquired with LSRII or Symphony A3 cytometers (BD Biosciences, Franklin Lakes, NJ, USA) and analyzed with FlowJo software (TreeStar, Ashland, OR, USA).

### 2.7. Mitochondrial Integrity

MitoTracker Green FM (Molecular Probes, Invitrogen, Waltham, MA, USA) was used to measure mitochondrial mass by flow cytometry analysis following the manufacturer’s instructions. Briefly, 5 × 10^5^ cells were collected and stained with MitoTracker Green (100 nM) for 45 min at 37 °C. Cells were washed with PBS, collected, and analyzed by flow cytometry.

To assess mitochondrial functional integrity, the mitochondrial membrane potential Δψ*m* of Treg cells was measured by flow cytometry analysis by adding the fluorescent probe Tetramethylrhodamine, ethyl ester (TMRE, Invitrogen, at 50 nM) to the cell culture for 30 min before immediate analysis. Each condition was tested with the corresponding control of specificity, pretreating the cells with the mitochondrial uncoupler trifluoromethoxy-carbonylcyanide-phenylhydrazone (FCCP, 5 μM, Sigma-Aldrich, St. Louis, MO, USA) for 10 min at 37 °C to eliminate any Δψ*m*-independent TMRE signal and ensure the specificity of the Δψ*m* measurements.

### 2.8. Autophagy

To compare autophagic responses in Treg cells exposed to AZD8055, RAPA, or no drug, we assessed autophagic vesicle formation and autophagic vesicle accumulation with the Cyto-ID Green detection reagent (Enzo Life Sciences, Farmingdale, NY, USA), following the manufacturer’s instructions. The formation of autophagosome vacuoles was assessed in 3 × 10^5^ cells, collected, and incubated in 200 μL of cell culture medium for 30 min with the Cyto-ID Green probe. The accumulation of autophagic vacuoles was measured in corresponding samples after the blockage of autophagolysosomal degradation by chloroquine (CLQ, Enzo Life Sciences, at 10 μM) for 6 h before the addition of Cyto-ID Green solution. For each condition, vacuole accumulation was quantified by subtracting the Cyto-ID MFI value of the sample without CLQ from the Cyto-ID MFI value of the sample with CLQ.

### 2.9. Mitochondrial Unfolded Protein Response (mitoUPR) Cell Stress Response Pathway

The regulation of the cell stress response pathway mitoUPR was determined by intracellular expression changes of the following mitochondrial proteins: the serine protease Caseinolytic Peptidase-P (CLPp), the chaperone Glucose-Regulated Protein 75 (GRP75), the histone deacetylase Sirtuin-3 (SIRT3), and the antioxidant manganese Superoxide Dismutase (SOD2).

### 2.10. Metabolic Characterization

Bioenergy profile analyses evaluated mitochondrial metabolism with oxygen consumption rates (OCR, pmol/min) and extracellular acidification rates (ECAR, mpH/min) in living Treg cells using the MitoStress test and the Glycolytic Rate Assay kits (Seahorse Bioscience, Agilent Technologies, Santa Clara, CA, USA), respectively. The assays were performed on an XF96 extracellular flux analyzer (Seahorse Bioscience, Agilent Technologies, Santa Clara, CA, USA) using the protocol and conditions optimized for primary T cells as reported [16]. The energy profile map is a scatterplot of OCR (horizontal axis) and ECAR (vertical axis) displaying the baseline phenotype, stressed phenotype (as the maximal potential values of both OCR and ECAR), and metabolic potential (as the measure of cellular ability to meet the energy demands by plotting the utilization of both pathways in response to metabolic stressors). Additional analyses were performed with Wave 2.2 software (Seahorse Bioscience), Excel (Microsoft Office 2019, Seattle, WA, USA), and PRISM 9.0 (GraphPad Software, San Diego, CA, USA).

### 2.11. Statistics

Immunological data was reported as mean ±SD. Experiments were performed in (at least) triplicate wells for each condition, and each experiment was repeated at least three times with samples obtained from different donors. Statistical differences were analyzed by one-way ANOVA with Dunnettt’s test or two-tailed Student’s *t*-test for pairwise comparisons. Analyses were performed using GraphPad PRISM 9.0.

## 3. Results

### 3.1. Isolation and Culture of CD4^+^CD25^+^ Treg Cells

We isolated Treg cells from leukapheresis product following our previously published method [15], and obtained a cell product with a purity above 85% of CD4^+^/CD25^HIGH+^/FOXP3^+^ cells and a viability greater than 90%.

**Figure 1:**
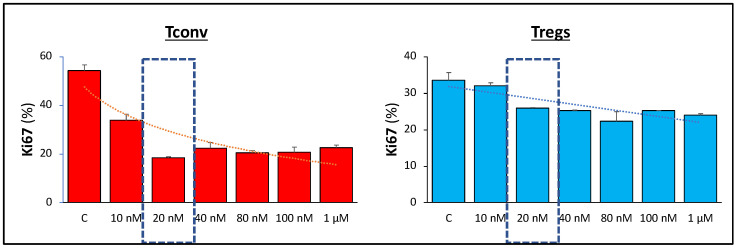
**Selection of effective AZD8055 concentration.** Equal numbers of CD25^+^-enriched cells (Tregs) and CD4^+^ T cells (Tconv) were cultured with different concentrations of AZD8055. After 24 h, cells were collected and the expression of the proliferation marker Ki67 was assessed by intracellular staining and flow cytometry analysis. The results are shown as mean ± SD (*n = 3*) of individual doses (bar graphs) and corresponding dose-response trendlines (dotted lines). Dashed window shows the dose of AZD8055 selected (20 nM) as the dose that induced maximum inhibition in Tconv cells (66.2% ± 8.4 inhibition) with low effects in Tregs (32.7% inhibition ± 7.3).

Since AZD8055 has not been previously used in Treg cell culture/expansion, we first determined the optimal concentration of AZD8055 in cell culture to selectively inhibit Tconv proliferation while minimizing the effects on CD25^+^ Treg cell expansion. Treg and homologous Tconv cells were exposed to increasing doses of AZD8055 ranging from 10 nM to 1 µM, and the expression of Ki67 nuclear protein was analyzed after 24 h as a proliferation marker. The results established 20 nM as the minimum effective dose of AZD8055 that promotes the maximum selective activity against Tconv (Figure 1). Accordingly, we set 20 nM as the default concentration of AZD8055 used in CD4^+^CD25^+^ Treg-enriched cells throughout the study.

### 3.2. Signaling

**Figure 2:**
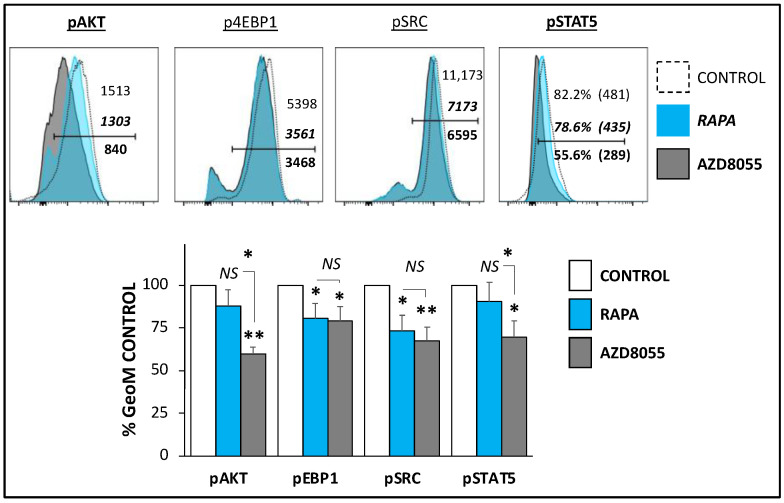
**AZD8055 in mTOR and IL2-R in Treg cell signaling.** Cells were expanded in Treg cell medium with vehicle control, RAPA (100 nM) or AZD8055 (20 nM). After 5 days, cells were collected and analyzed by flow cytometry for the expression of the activated forms of the mTORC2 substrate, AKT (pAKT); the mTORC1 substrate 4EBP1 (p4EBP1); the IL2-R-dependent phosphorylation of STAT5 (pSTAT5); and the activated form of SRC kinase (pSRC). The top panels show representative histogram panels with the expression profiles of vehicle control (dashed, empty histograms)-, RAPA (blue-filled)-, and AZD8055 (grey filled histograms)-treated Treg cells. Each panel includes the GeoMFI values (pSTAT5 also percentages of positive cells) for each condition. Control values are shown in regular font, RAPA-treated in italic bold and AZD8055-treated in standard bold font. The bottom bar panel illustrates the comparative average protein expression ± SD pooled from three different experiments *(n = 3)*. The expression of each individual protein is depicted as a percentage of its corresponding control (untreated) condition. * *p* < 0.05 and ** *p* < 0.001 indicate significant differences identified by one-way ANOVA, followed by Dunnett’s post hoc test. *NS* indicates no significant differences between treatments.

AZD8055 is an ATP-competitive inhibitor of mTOR kinase activity [21]. We compared the signaling changes induced in Treg cells after 30 h culture in the presence of AZD (20 nM) compared to those in RAPA at the standard concentration used for Treg cell expansion (100 nM). Flow cytometry analyses of homologous cells showed significant differences between both mTOR protein kinase inhibitors in the expression levels of activated AKT, as measured by phosphorylation of S^473^, a recognized downstream target of mTORC-2 kinase. In contrast, the cells showed similar expression of the mTORC1 target phospho-4EBP1 expression. These results indicate the different impact of RAPA and AZD8055 treatments on mTORC1/mTORC2 balance in Treg cells. In contrast, no significant differences were noted in the activation of SRC, indicative that proximal T cell antigen receptor signal transduction (including the activation of Lck and Fyn kinases) [22] was not differently targeted by RAPA and AZD. However, the IL2-receptor-triggered STAT5 signaling (measured by phosphorylated form of STAT5 expression levels) was significantly weaker in AZD-treated Treg cells compared with those treated with RAPA. Altogether, these results show that RAPA and AZD8055 induce different signaling activation patterns in Treg cells (Figure 2).

### 3.3. Proliferation and Cell Viability

**Figure 3:**
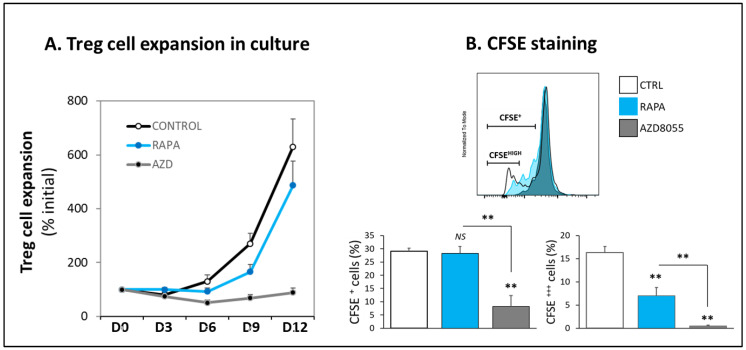
**AZD8055 in Treg cell expansion.** (**A**) Long-term effect of RAPA (100 nM, blue symbol and blue line) and AZD8055 (20 nM, black symbol and grey line) compared to control vehicle-treated cells (white symbol, black line) on the expansion of Treg cells. Addition of the selective dose of AZD8055 (20 nM) to the cell culture media prevents long-term expansion of clinically competent Treg cells. (**B**) CFSE labeling was used to monitor Treg cell divisions; dividing cells were identified as sequential diming of CFSE fluorescent intensity. After 5-day culture, AZD8055-treated Treg cells (grey-filled histogram and bars) were not dividing. In contrast, the percentage of RAPA-treated cells (blue-filled histogram and bars) was similar to that of the untreated control cells (unfilled histogram and bars), although the number of divisions per cell was substantially lower, as illustrated by the reduced percentage of CFSE^DIM^ cells compared with untreated cells (right bar panel). ** *p* < 0.001 indicate significant difference as measured by one-way ANOVA and Dunnett’s test. *NS* differences were not significant (*p* > 0.05).

During a 12-day expansion period, Treg cells treated with AZD8055 did not undergo substantial proliferation. In contrast, as anticipated, RAPA-treated cells exhibited a 5-fold expansion, while the vehicle control cells expanded 6-fold after 12 days under optimized Treg culture conditions (Figure 3A). CFSE labeling showed that 4-day AZD8055-treated Treg cells were not dividing, while the percentage of homologous CFSE^+^-proliferating RAPA-treated cells was similar to the corresponding untreated cells, although, as expected, RAPA treatment reduced the number of divisions per cell, as illustrated in the lower percentage of CFSE^DIM^ (Figure 3B).

Despite these different proliferative responses, no significant differences in cellular viability were shown among RAPA, AZD8055, and untreated cells after 4-day cell culture (Figure S1).

### 3.4. Suppressive Function

**Figure 4:**
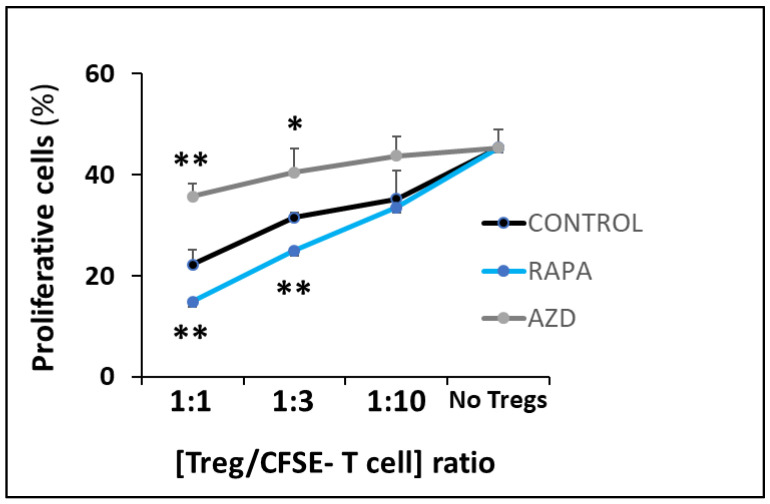
**AZD8055 in Treg cell function.** Loss of suppressor activity in peripheral Treg cells after 3-day-exposure to AZD8055 (grey dots) compared to RAPA-treated (blue dots) or untreated (control) cells (black dots). Suppressor function of Treg cells was determined by the inhibition of CFSE-labeled T cell expansion in a 4-day mixed lymphocyte reaction with different Treg/CFSE-labeled cell ratios. Results are shown as mean ±SD of proliferative cells evaluated by flow cytometry from three independent experiments. * *p* < 0.05 and ** *p* < 0.001 indicate significant differences determined by one-way ANOVA and Dunnett’s post-hoc test.

In addition to the inability to support extended cell expansion, AZD treatment in Treg cells resulted in a significant loss of suppressor activity, in sharp contrast with the enhanced regulatory function induced by RAPA (Figure 4). Overall, these results depict the substantial adverse effects of AZD treatment on Treg cell function.

### 3.5. Mitochondrial Morphology and Function

**Figure 5:**
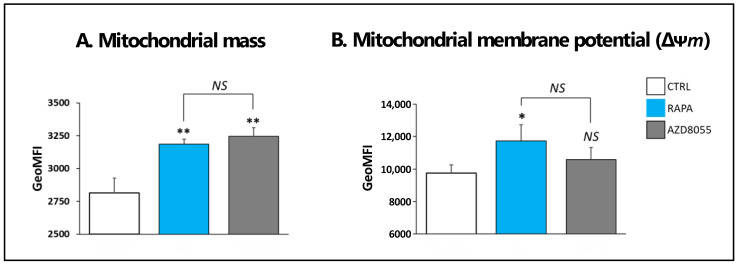
**AZD8055 in mitochondrial integrity.** (**A**) MitoTracker Green FM (Molecular Probes, Invitrogen) was used to measure mitochondrial mass by flow cytometry analysis. Results are expressed as mean fluorescence intensity (MFI) and show the significant increase of mitochondrial mass in AZD- and RAPA-treated cells compared to untreated, control cells, but no differences between the effects induced by the drugs. (**B**) Δψ*m* was measured by flow cytometry analysis with TMRE (Invitrogen) staining. Each condition was tested with the corresponding control of specificity by pre-treatment of cells with the mitochondrial uncoupler FCCP (5 µM) to ensure the specificity of the Δψ*m* measurements. The results show a significant Δψ*m* increase only in RAPA-treated and not AZD-treated cells. ** *p* < 0.001 and * *p* < 0.05 indicate significant differences as measured by one-way ANOVA and Dunnett’s test. *NS*: no significant differences.

We next addressed the potential mechanisms of action of AZD in Treg cells. In a previous study, we reported the enhancement of mitochondrial mass in T cells after RAPA treatment, with the corresponding increase in mitochondrial membrane potential (∆ψ*m*), a critical parameter of mitochondrial functional integrity [16]. Consistent with these results, Treg cells exposed to RAPA for 30 h resulted in increased mitochondrial mass compared with untreated cells, to the same extent as AZD-treated cells (Figure 5A). However, unlike RAPA treatment, AZD exposure did not result in any significant Δψ*m* change, as denoted by similar shifts of fluorescence intensity peak measurements in TMRE/FCCP labeled control (no drug) and AZD-treated cells (Figure 5B). These results may count as the first indicators of AZD-induced functional stress in Treg mitochondrial function and suggest that AZD treatment promoted an imbalance between mitochondrial biogenesis and degradation.

### 3.6. Autophagic Response

**Figure 6:**
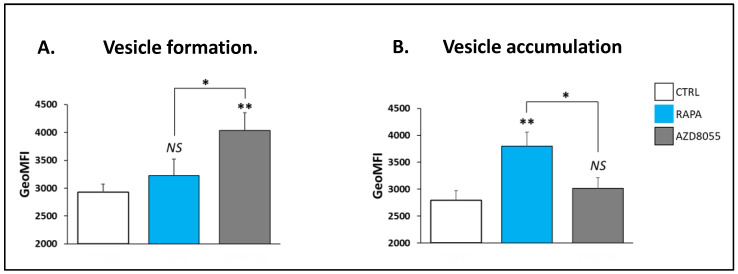
**AZD8055 in autophagy.** Autophagy responses were monitored with flow cytometry analysis using the Cyto-ID Green detection reagent (Enzo Life Sciences). (**A**) The enhancement of Cyto-ID Green dye signal (MFI) indicates a higher increase in autophagosome vacuole formation in AZD-treated cells compared with RAPA-treated cells. (**B**) The accumulation of autophagic vacuoles was measured after the blockage of autophagolysosomal degradation by Chloroquine (CLQ, 10 μM) for 6 h before the addition of Cyto-ID Green solution. Vacuole accumulation was quantified by subtracting the Cyto-ID MFI value of the sample without CLQ from the Cyto-ID MFI value of the sample with CLQ for each condition. Unlike RAPA treatment, addition of AZD did not induce the accumulation of autophagosomes in Treg cells, which, together with a higher autophagosome formation, is consistent with impaired autophagic flux. * *p* < 0.05 and ** *p* < 0.001 indicate a significant difference as measured by one-way ANOVA followed by Dunnett’s test. *NS* differences were not significant (*p* > 0.05).

It is well established that the activation of the PI3K/mTOR pathway negatively regulates autophagy and that the mTOR inhibitor RAPA promotes autophagy in different cell settings [16,23,24,25]. We asked whether the addition of another selective mTOR kinase inhibitor, AZD8055, in the cell culture induced similar effects in Treg cells. The analyses with the Cyto-ID Green detection reagent demonstrated that AZD increased the formation of autophagosome vacuoles to a greater extent than RAPA treatment (Figure 6A). In contrast, the accumulation of autophagic vesicles (a measure of autophagic degradation potential) was significantly reduced compared with that of RAPA-treated Treg cells (Figure 6B). These results are consistent with a progressive AZD-induced deterioration of autophagic response secondary to an impaired autophagic flux.

### 3.7. mitoUPR Changes

**Figure 7:**
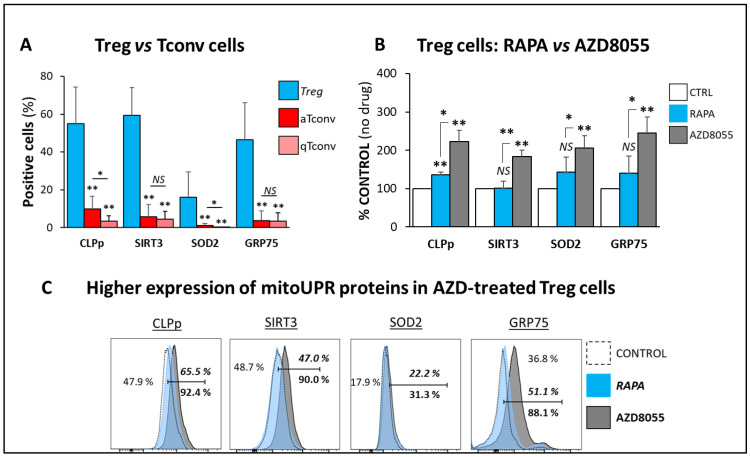
**AZD8055 in mitoUPR response.** The regulation of mitochondrial unfolded protein response (mitoUPR) was determined by the analysis of the protein expression levels of mitochondrial proteins Caseinolytic peptidase-P (CLPp), the chaperone Glucose-Regulated Protein 75 (GRP75), Sirtuin-3 (SIRT3) and the antioxidant manganese Superoxide Dismutase (SOD2). (**A**) The expression of all mitoUPR proteins was measured from five different biological samples of freshly isolated Treg cells compared to activated (CD25^+^) or quiescent (CD25^−^) conventional T cells. Pooled results demonstrated that the expression of all mitoUPR proteins was significantly higher in Treg cells (blue-filled bars) compared with activated (aTconv, red-filled bars) and quiescent (qTconv, maroon-filled bars). (**B**) The expression of mitoUPR proteins in Treg cells show increased fluorescent intensity of all mitoUPR expression proteins tested in Treg cells cultured in the presence of AZD8055 for 48 h (grey-filled bars) compared with RAPA cultured cells (blue-filled bars) and control vehicle-treated (unfilled bars) measured from three biological samples. Pooled results demonstrated that the expression of all mitoUPR proteins was significantly higher in AZD-treated compared to RAPA-treated and control cells. (**C**) Representative flow cytometry histogram overlay plot showing increased fluorescent intensity of all mitoUPR expression proteins tested in Treg cells cultured in the presence of AZD8055 for 48 h (grey-filled histograms) compared with RAPA cultured cells (blue-filled histograms) and control (vehicle) cells (dashed, empty histograms). Intra-panel numbers show percentages of positive control cells in regular font, RAPA-treated in italic, and AZD-treated in standard font. * *p* < 0.05 and ** *p* < 0.001 indicate significant difference between treatment conditions determined by one-way ANOVA and Dunnett’s post-hoc test. *NS* differences did not reach the levels of significance (*p* > 0.05).

The significant changes in the autophagic mechanisms in AZD8055-treated cells prompted us to investigate the impact of AZD on the regulation of mitoUPR, a protective cellular stress response pathway that prevents mitochondrial dysfunction in proteotoxic and/or metabolic stress conditions by inducing the expression of mitochondrial chaperones and proteases in response. Here, we provide initial evidence on the higher basal expression level of all mitoUPR-related proteins measured in Treg cells compared with Tconv cells (Figure 7A). These results constitute, to our knowledge, the first experimental evidence of the distinct potential role of this adaptive stress response system in the regulation of mitochondrial integrity in Treg cells. Further supporting the induction of mitochondrial stress by AZD8055 in Treg cells, the same AZD treatment accounted for suboptimal functional mitochondrial integrity and led to the overexpression of all mitoUPR proteins tested, as determined by the higher fluorescent intensity readings in CLPp, SIRT3, SOD2, and GRP75 compared with RAPA-treated Treg cells (Figure 7B). These results revealed the elevated basal levels of mitoUPR expression in Treg cells and the overactivation of this pathway in response to stressful stimuli.

### 3.8. Cell Metabolism and Bioenergetics

**Figure 8:**
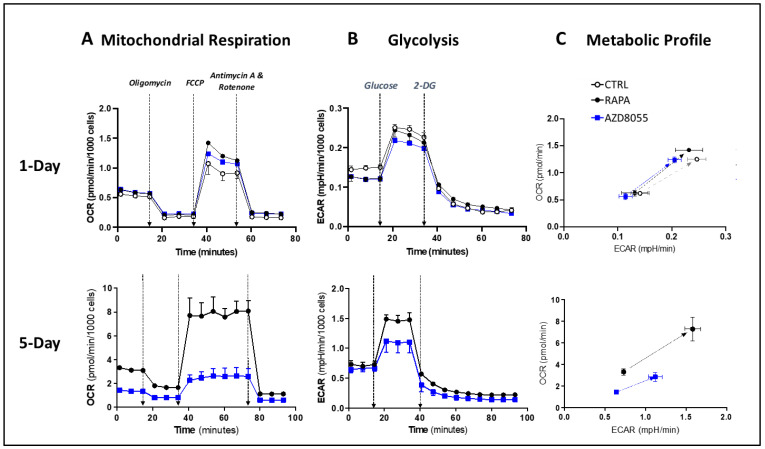
**AZD8055 in Treg cell bioenergetics.** Mitochondrial respiration (measured by oxygen consumption rate, OCR, panels **A**), glycolytic function (measured by extracellular acidification rate, ECAR, panels **B**), and energy profile maps (panels **C**) were analyzed in purified Treg cells treated with AZD8055 (20 nM, square blue symbols), RAPA (100 nM, black filled circles), or untreated control (empty circles) for 1 day (**top panels**) or 5 days (**bottom panels**). Metabolic profile illustrates the metabolic potential of RAPA- or AZD-treated Treg cells from baseline phenotype to the maximum metabolic (OCR and ECAR) responses as the measure of the cellular ability to meet the energy demands by plotting the utilization of both pathways in response to metabolic stressors. Metabolic profile of Treg cells after 1-day culture shows similar metabolic potential in untreated (control) and RAPA- or AZD-treated Treg cells. In contrast, by day 5, AZD treatment has caused a significant metabolic deterioration in Treg cells compared with RAPA. These results are consistent with the metabolic inability of AZD-treated cells to meet the energy demands of a functionally competent Treg cell. Data represent one of at least three experimental (biological) replicates with at least five technical replicates each (multiple measurements).

To evaluate the effects of AZD on the bioenergetic status of the Treg cells, we measured oxygen consumption rates (OCR, a measure of mitochondrial respiration) and extracellular acidification rates (ECAR, an indicator of glycolytic cell function). In the early phase of drug exposure (1-day culture), mitochondrial respiration demonstrated similar sensitivity to AZD and RAPA, while the alteration of the glycolytic pathway was more noticeable in AZD-treated than in RAPA-treated cells (Figure 8 top panels and Figure S2). After 5-day exposure, the differences between the mTOR inhibitor treatments were steep. AZD-treated T cells exhibited a prominent reduction in both OCR and ECAR (Figure 8 left and central bottom panels and Figure S2). The progression of the cellular metabolic profiles illustrates the lower bioenergetic potential of the 5-day AZD-treated cells compared with that of 1-day AZD-treated cells and 5-day RAPA-treated cells (Figure 8C). These results are consistent with a significant functional mitochondrial deterioration and support the inability of AZD-treated cells to meet the energy demands of functionally competent Treg cells.

## 4. Discussion

Optimization of ex vivo expansion is a crucial step in current protocols for manufacturing a final cell product of polyclonal and antigen specific Treg cells with consistent cell yield, viability, and efficacy adequate for therapeutic use. Low viability, loss of suppressive function, and contamination risks are some of the challenges associated with prolonged cell culture and manipulation. To address these challenges, our group and others are investigating alternative methods to improve the Treg cell manufacturing process by shortening expansion time while maintaining or improving cell viability and suppressive function. The employment of RAPA to regulate the mTOR signaling pathway is an initial attempt to enhance the purity of the final Treg cell product by selective inhibiting Tconv proliferation. We have previously studied the use of Everolimus as an alternative to RAPA for ex vivo expansion of Treg cells. Our results demonstrated that the drugs induced different patterns of mTOR signaling inhibition associated with temporary initial slower growth and lower metabolic rates in Everolimus-treated cells, but similar long-term expansion and suppressive function [16]. We have also explored agents with dual PI3K and mTOR inhibition properties such as PI-103 and PKI-587 on Treg cell expansion and demonstrated that targeting mitochondrial respiration resulted in major disruption of Treg cell suppressive function [17]. AZ8055 is a strong mTORC1/C2 inhibitor originally developed to treat hematologic malignancies [21]. In this study, we compared the efficacy of this potent mTORC1/C2 inhibitor with RAPA on Treg cell ex vivo expansion for clinical use. As expected, we found a stronger inhibition of the mTORC2 signaling pathway (determined by AKT phosphorylation), but also of downstream events triggered by IL2-receptor engagement (assessed by phosphorylation levels of STAT5) in AZD8055-treated cells compared with RAPA-treated cells (Figure 2). Although cell viability was not initially affected (Supplementary Figure S1), the exposure to AZD8055 interfered with signaling evets in Treg cells that disrupt long-term expansion and functional integrity of the cell (Figure 3 and Figure 4). These results confirm the high sensitivity of the mTORC1/C2 balance to sustain the activation and functional state of Treg cells and demonstrate the unsuitability of AZD8055 for ex vivo manufacturing clinically competent Treg cells. Since the differential loss of mTORC2 exerts such substantial disruption of Treg activity, we next studied cellular mechanisms triggered in AZD8055-treated cells compared to RAPA-treated cells.

We demonstrated that both drugs promoted similar increases in mitochondrial mass, a phenomenon reportedly promoted by in RAPA in Treg and other cell types [16,17,26]. In our previous studies, the enhanced mitochondrial mass in RAPA-treated Treg cells was linked to hyperpolarization of the mitochondrial membrane, increase in autophagy flux, and higher cell oxidative metabolism and glycolytic capacity [16,17]. However, in the current study, the changes in mitochondrial mass induced by AZD8055 treatment did not involve a corresponding increase in mitochondrial membrane potential (Figure 5), suggesting an early loss of mitochondrial function efficacy consistent with a status of functional mitochondrial stress [27]. Our results also revealed that AZD8055 treatment promoted early autophagosome formation but lowered autophagosome accumulation upon autolysosome inhibition (Figure 6). A similar pattern of disturbed autophagic flow has been reported in oxidative-stress mitochondrial damage induced in vascular endothelial cells [28], which leads to the inability of eliminating non-functional organelles. In a previous study, we showed that the accumulation of dysfunctional mitochondria may result in the progressive deterioration of Treg cell activity and fitness [19].

These results further suggest that AZD8055 treatment drives Treg cells to a significant state of mitochondrial stress. In support of this premise, we evaluated mitoUPR activation, which has been associated with an early-phase response to mitochondrial stress to prevent cellular damage and preserve cell homeostasis. Our results identified higher baseline mitoUPR levels in Treg cells compared with Tconv (Figure 7A), likely consequent to an elevated mitochondrial oxidative metabolism, higher mitochondrial mass, and greater oxidative stress potential of Treg cells [17,29,30]. Proper regulation of mitochondrial function with the involvement of mitoUPR has been described as essential for adequate Tconv cell activation, differentiation, survival, and effector activity [30]. Still, the role in Treg cells has not been yet defined. Remarkably, we found increased expression levels of all mitoUPR components tested (CLPp, SIRT3, SOD2, and GRP75) in AZD8055-treated compared with RAPA-treated Treg cells (Figure 7B). We hypothesize that AZD8055 treatment enhanced mitoUPR activity as a stress response to prevent mitochondrial proteotoxic damage in Treg cells. However, long-term exposure to AZD8055 alters the protective effects of mitoUPR and autophagy, leading to the accumulation of dysfunctional mitochondrial proteins and intracellular organelle deterioration with subsequent disruption of Treg cell function.

Since the mTOR pathway is a critical sensor of the cell metabolic status and driver of the metabolic cell plasticity, the pharmacological metabolic reprogramming through mTOR targeting constitutes an attractive approach to control the Treg/Tconv cell homeostasis [16,17,18,19,20,29,31,32,33]. Our previous studies supported an effective pharmacological range of mTOR targeting that achieves the desired selective and functional effects on Treg cells that correlates with satisfactory manufacturing of clinical grade Treg cells [16]. However, subtle quantitative and qualitative drug changes can exceed the threshold of pharmacological range efficacy [17,18]. In this study, we observed that AZD8055-treated Treg cells undergo a metabolic reprogramming that drives cells to a functionally quiescent status (Figure 8). Mitochondrial dysfunction associated with impaired autophagy and the inability to meet cellular bioenergetic requirements could explain, at least in part, the observed loss of proliferative potential and suppressive function in AZD8055-treated Treg cells.

## 5. Conclusions

AZD8055 promoted a distinctive inhibition of the mTOR signaling pathway that resulted in reduced Treg cell proliferation and impaired suppressive function, which precludes the use of AZD8055 in the manufacturing process of clinically competent Treg cells. This study depicts a tightly coordinated and integrated adaptive network of signaling, cellular stress responses, and metabolic pathways in Treg cells triggered by the disruption of the mTOR-dependent cellular homeostasis. Table 1 presents a concise overview of the differing effects of RAPA and AZD8055 on Treg cells reported in this research.

The new finding of mitoUPR involvement in the regulation of Treg cell function and fate warrants further investigation, not only as an early indicator of Treg mitochondrial stress status, but also as a promising potential target for therapeutic interventions.

## Figures and Tables

**Table 1:** Differential effects promoted by RAPA and AZD8055 in Treg cells.

	Target	Rapamycin	AZD8055
**Signaling**	mTORC1	↓	↓
	mTORC2	◯	↓
	IL2-R pathway	◯	↓
**Cell function**	Expansion	↓	↓↓
	Suppressor Activity	↑	↓
**Mechanism of action**	Mitochondrial stress	◯	↑
	Autophagy	↑	↓
	mitoUPR	◯/↑	↑↑
	Cell bioenergetics	◯	↓↓

Relative effects compared to vehicle (control) treated cells: ↓ inhibitory effect; ◯ equivalent effect; ↑ enhancing effect.

## Data Availability

Data relevant to the study are included in the article or uploaded as Supplementary Information. All data generated in this study are available upon reasonable request from the corresponding author.

## Supplementary Materials

The following supporting information can be downloaded at: https://www.mdpi.com/article/10.3390/cells12162066/s1, Table S1: List of fluorochrome-conjugated antibodies used in this study; Figure S1: Treg cell viability; Figure S2: Cell bioenergetic parameters.

## Abbreviations

PE, phycoerythrin; PerCP-Cy5.5, Peridinin chlorophyll protein-Cyanine5.5; FITC, fluorescein isothiocyanate; eF, eFluor; APC, allophycocyanine; PE-Cy7, PE-Cyanine7; Cat#, Catalog Number; RRID, Research Resource Identifier.
