# Supplementary material for: Metabolic Disruption Induced by mTOR Signaling Pathway Inhibition in Regulatory T-Cell Expansion for Clinical Application
Source: Cells. 2023 Aug 15;12(16):2066. doi: 10.3390/cells12162066 (PMC10453008; doi:10.3390/cells12162066)
Supplement: Supplementary file 1 [file cells-12-02066-s001.zip › cells-2541377-supplementary.pdf]

cells

MDPI

SUPPLEMENTARY MATERIALS

Table S1: List of fluorochrome-conjugated antibodies used in this study.

Antibody	SOURCE	IDENTIFIER
Anti-CD127 (PE)	BioLegend	Cat #: 351340; RRID: AB_2564136
Anti-CD25 (PE)	BioLegend	Cat #: 356134; RRID: AB_2564145
Anti-CD25 (PerCP-Cy5.5)	BioLegend	Cat #: 356112; RRID: AB_2561979
Anti-CD3 (BrilliantViolet-421)	ThermoFisher	Cat #: 404-0037-42; RRID: AB_2925485
Anti-CD3 (FITC)	ThermoFisher	Cat #: 11-0038-42; RRID: AB_2043831
Anti-CD4 (APC eF780)	ThermoFisher	Cat #: 47-0049-42; RRID: AB_1272044
Anti-CD4 (FITC)	ThermoFisher	Cat #: 11-0049-42; RRID: AB_1659694
Anti-CD4 (PerCP-Cy5.5)	ThermoFisher	Cat #: 45-0049-42; RRID: AB_1518744
Anti-CD8 (APC-Fire780)	ThermoFisher	Cat #: 47-0088-42; RRID: AB_1272046
Anti-CD8 (PE-Cy7)	ThermoFisher	Cat #: 25-0088-42; RRID: AB_1659702
Anti-FOXP3 (eF660)	ThermoFisher	Cat #: 50-4776-42; RRID: AB_10597604
Anti-FOXP3 (PE)	BioLegend	Cat #: 320208; RRID: AB_492982
Anti-Ki67 (BriliantViolet-510)	BioLegend	Cat #:151225 RRID: AB_2722785
Anti-phospho-S^{473}-AKT (PE)	ThermoFisher	Cat #: 12-9715-42; RRID: AB_2637101
Anti-phospho-$T^{36/45}$-4EBP1 (PE)	ThermoFisher	Cat #:12-9107-42 RRID: AB_2572693
Anti-phospho-Y^{694}-STAT5 (PE)	ThermoFisher	Cat #: 12-9010-42 RRID: AB_2572671
Anti-phospho-Y^{418}-SRC (eF660)	ThermoFisher	Cat #: 50-9034-42; RRID: AB_2572681
Anti-CLPp (AlexaFluor-488)	Santa Cruz Biotechnology	Cat #: sc-271284; RRID: AB_10610081

Anti-SOD2 (AlexaFluor-488)	Santa Cruz Biotechnology	Cat #: sc-133134<br>RRID: AB_2191814
Anti-SIRT3 (PE)	Santa Cruz Biotechnology	Cat #: sc-365175;<br>RRID: AB_10710522
Anti-GRP75 (AlexaFluor-647)	Santa Cruz Biotechnology	Cat #: sc-133137;<br>RRID: AB_2120468

Abbreviations: PE, phycoerythrin; PerCP-Cy5.5, Peridinin chlorophyll protein-Cyanine5.5; FITC, fluorescein isothiocyanate; eF, eFluor; APC, allophycocyanine; PE-Cy7, PE-Cyanine7. Cat #, Catalog Number; RRID, Research Resource Identifier.

Supplementary Figures

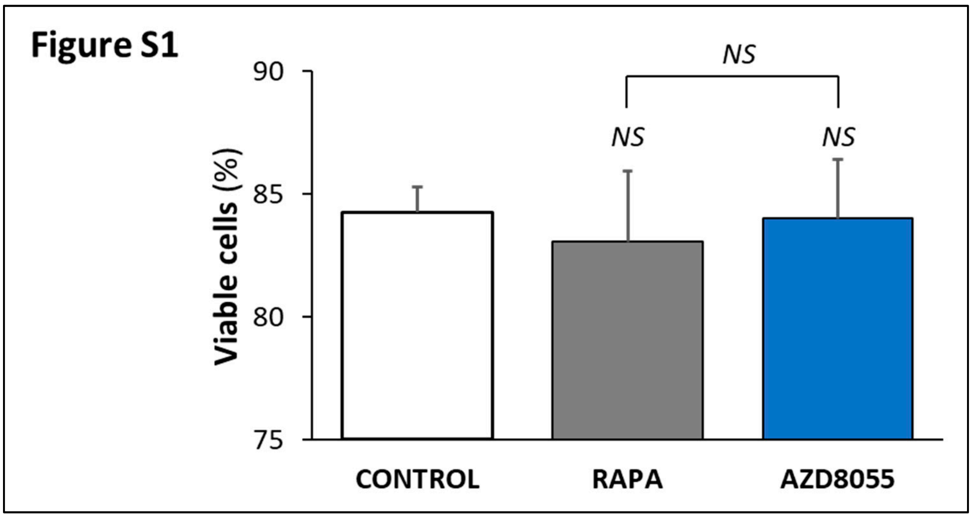

Figure S1- **Treg cell viability**. After 5 days in Treg cell culture medium with vehicle control or with RAPA (100 nM) or AZD8055 (20 nM), Treg cells were collected and labeled with the Zombie Violet Fixable Viability kit (BioLegend). The percentages of viable (unstained) cells were measured by flow cytometry analysis. Bar graphs show the pooled average ± SD data from 3 different experiments (n=3). No significant (NS) differences were found in cell viability between untreated (control) and RAPA- or AZD-treated cells as determined by one-way ANOVA test.

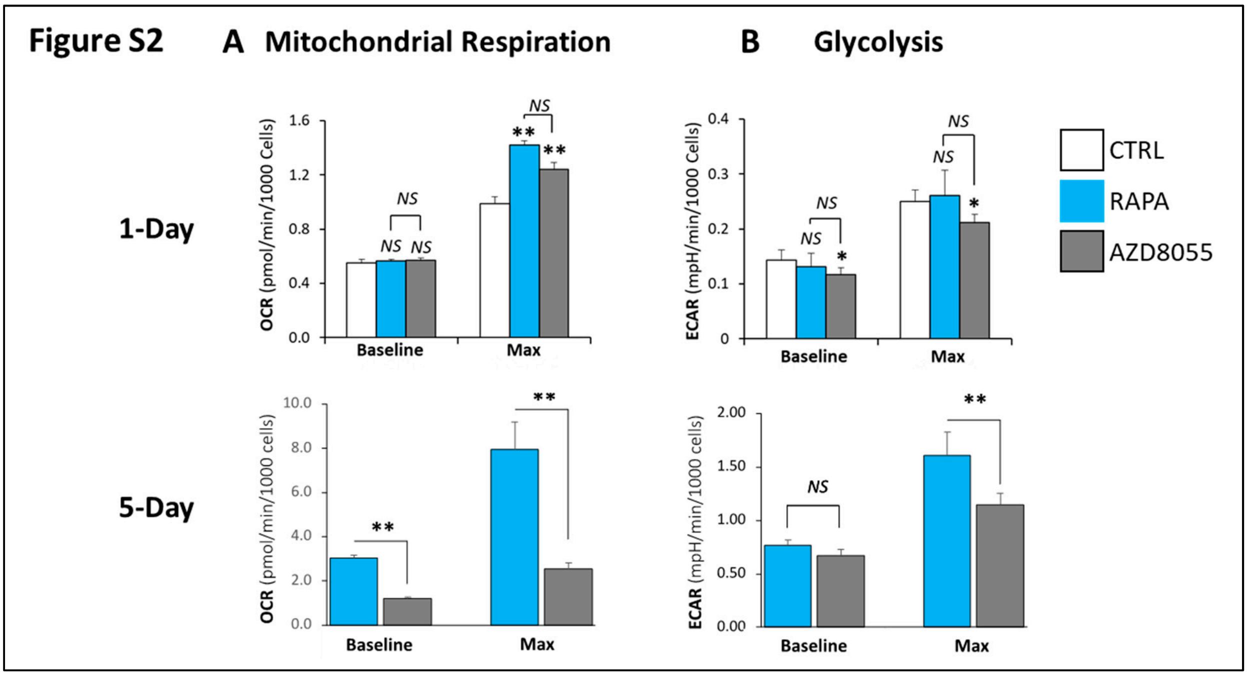

Figure S2 (Supplementary to Figure 8 of the Main Text). **Bioenergetic parameters** of Treg cells in cell medium with vehicle control (untreated cells) or in the presence of RAPA (100 nM) or AZD8055 (20 nM) for 1 day (top panels) or 5 days (bottom panels). Bar graphs show baseline and maximum levels of parameters measured from the corresponding OCR or ECAR profiles and were generated from the Seahorse XF Mito Stress Test Report Generator and the Glycolysis Report Generator, respectively. Data represents the mean ± SD pooled from at least three independent *(n=3)* experiments and five replicates per condition in each experiment. *p<0.05 and **p<0.001 indicate significant differences among cell culture treatments as measured by one-way ANOVA and post-hoc Dunnett's test. *NS* indicates no significant differences.
